# Characteristics of health care workers with SARS-CoV-2 at a COVID-19 hospital in Türkiye: Homologous versus heterologous vaccination

**DOI:** 10.12669/pjms.40.8.8455

**Published:** 2024-09

**Authors:** Isıl Deniz Alıravcı, Yusuf Haydar Ertekin, Gamze Can, Sevil Alkan

**Affiliations:** 1 Işıl Deniz Alıravcı, MD Assistant Professor, Department of Infectious Diseases and Clinical Microbiology, Canakkale Onsekiz Mart University, Canakkale, Türkiye; 2 Yusuf Haydar Ertekin, MD Associate Professor, Department of Family Medicine, Canakkale Onsekiz Mart University, Canakkale, Türkiye; 3 Gamze Çan, MD Professor, Department of Public Health, Canakkale Onsekiz Mart University, Canakkale, Türkiye; 4 Sevil Alkan, MD Associate Professor, Department of Infectious Diseases and Clinical Microbiology, Canakkale Onsekiz Mart University, Canakkale, Türkiye

**Keywords:** COVID-19, Health personnel, COVID-19 vaccine, SARS-CoV-2, Vaccination, BNT162 Vaccine

## Abstract

**Objective:**

Given the limited studies on types of vaccination and infection rates among health care workers (HCWs) in Türkiye, we analyzed the demographic, clinical, and vaccination characteristics as well as the attitudes of HCWs who have been infected with COVID-19.

**Methods:**

We retrospectively analyzed demographic and clinical data on breakthrough COVID-19 infections in HCWs from hospital surveillance data collected between April 5, 2020, and November 1, 2022. The comparison was based on four subgroups that consisted of unvaccinated, one-shot-vaccinated, homologous vaccinated, and heterologous vaccinated individuals. Participants who received various combinations of Sinovac/CoronaVac and/or BioNTech/Pfizer vaccines were compared for detection of COVID-19.

**Results:**

During a 33-month period of 744 HCWs who contracted COVID-19, women (65.3%) and nurses (28.9%) were the most affected, followed by doctors (25.8%). Of the infected HCWs, only 1.3% required hospitalization, 0.3% required ICU support, and 98.4% were outpatients. By vaccination status, 143 of the HCWs (19.2%) were unvaccinated, 292 (39.2%) were homologously vaccinated, 294 (39.5%) were heterologously vaccinated, 15 (2%) received a single shot, 206 (27.7%) received two shots, and 165 (22.2%) received three shots. All HCWs contracted COVID-19 at a mean of 134-days (range:1-539) after vaccination. While the proportions of homologously and heterologously vaccinated HCWs were similar, the time elapsed from vaccination to contracting COVID-19 varied (mean 143.4±106.7 vs.126.4±82.43 days).

**Conclusions:**

Among both outpatients and inpatients with COVID-19, women HCWs outnumbered men HCWs. HCWs who received homologous vaccination contracted COVID-19 later than those who received heterologous vaccination.

## INTRODUCTION

In Türkiye, by the end of November 2022, 17,042,722 cases of COVID-19 with 101,492 deaths had been reported^1^. According to the data of the National Ministry of Health, as of December 19, 2022, an estimated 152,596,243 COVID-19 vaccine doses have been administered in the country, and 28,226,697 (18%) of the population has received a three-shot course of the vaccine.^1^ Simultaneously in our hospital as in Türkiye’s COVID-19 vaccination program was launched across the nation on January 14, 2021, first with Sinovac/CoronaVac and later with BioNtech’s Messenger RNA BNT162b2 on April 2, 2021. Turcovac was implemented in July 2021. In our COVID-19 hospital, however, Turcovac has remained unavailable, so public workers and HCWs have been vaccinated only with the Sinovac/CoronaVac and BioNTech/Pfizer vaccines.

Recent data suggest that, beginning 14-days after the second dose, both the Sinovac/CoronaVac and BioNTech/Pfizer vaccines are effective in preventing symptomatic and severe COVID-19 in HCWs as well as hospitalization.^2^-^5^ However, given the emergence of new types of vaccines and debates about vaccine efficacy, HCWs have been vaccinated with homologous and heterologous types of vaccines. Therefore, data on vaccine efficacy for HCWs in Türkiye remained somewhat unclear. In response, we conducted a retrospective study to assess the incidence of SARS-CoV-2 infection among vaccinated and unvaccinated HCWs in Türkiye and to characterize HCWs who had developed COVID-19 in terms of their demographics, clinical characteristics, and vaccination status.

## METHODS

In our retrospective observational study, the HCWs’ cases of COVID-19-infection were all documented between April 6, 2020, and November 1, 2022, at Çanakkale Onsekiz Mart University Hospital. Demographic and clinical data regarding the HCWs’ cases of infection, including positive severe acute respiratory syndrome coronavirus-2 (SARS-CoV-2) PCR tests of swab samples and clinical findings together with positive chest tomography findings, were obtained from the hospital’s surveillance data starting from the first case. The HCWs whose swab samples were negative for SARS-CoV-2 were excluded.

The number of all hospital personnel working during the period of the study was 1538. Of these, 118 are faculty members, 271 are assistant doctors, and 425 are nurses and midwives. The remaining personnel -the group of “Other” are secretaries, security, cleaners, technicians and morgue workers. They were included in the study as they carried the risk of infection in the pandemic hospital.

**Fig.1:**
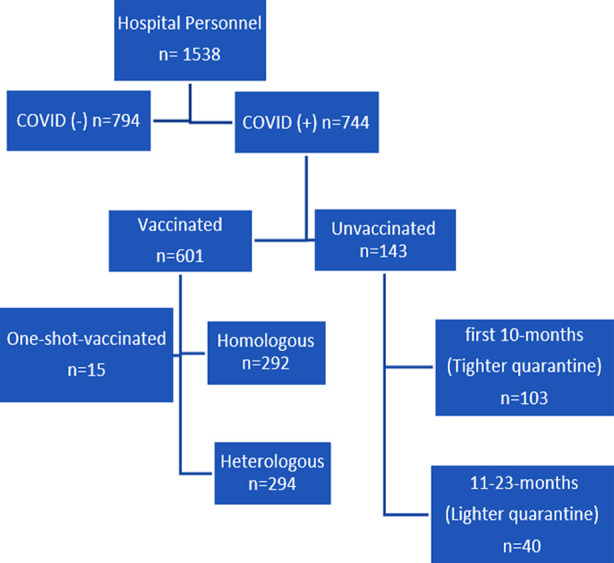
Study participation flow chart.

Covid-19 infected hospital personnel in the surveillance records kept by infection control committee nurses between 6 April 2020 and 1 November 2022 were included in the study. Only 744 COVID-19 infected hospital personnel recorded in the surveillance registry system as patients between these dates were included in the study, while 794 hospital personnel who were not infected were excluded from the study (Fig.1).

### Eligibility Criteria and Data Collection:

According to the course of COVID-19, the participants were classified into three groups: outpatients, inpatients (i.e., followed up in the COVID-19 ward), and intensive care unit (ICU) patients (i.e., followed up in the ICU). Vaccination status and vaccine doses of the participants were compared. We classified the vaccines as offering homologous, heterologous, or partial vaccination and tabulated the 14 different vaccine combinations administered to the HCWs.

The type of vaccination and the number of doses of vaccine administered were also considered. “Unvaccinated individuals” were defined as patients who had not received any vaccine dose. Patients who became symptomatic in less than two-weeks after receiving the second dose were labeled “Partly vaccinated.” Whereas patients labeled “Homologously vaccinated” were vaccinated with at least two doses of either the Sinovac/CoronaVac vaccine or the BioNTech vaccine, patients labeled “Heterologously vaccinated” were vaccinated with the Sinovac/CoronaVac and BioNTech vaccines, at one dose each.

### Statistical Analysis:

Data were evaluated with SPSS (SPSS Inc., Chicago, IL, USA,) version 26. Pearson’s chi-square test was used for categorical data, Fisher’s exact test was applied when appropriate, and the two-sample Student’s *t* test was used for the continuous variables. For categorical variables, the chi-squared or Fisher’s exact test was used along with ANOVA, Kruskal-Wallis variance analysis, or an independent sample *t* test wherever applicable. All *p* values were two-sided and considered to be statistically significant if less than 0.05.

Descriptive statistics of variables such as mean, median, standard deviation values, and frequency values were calculated. The data were evaluated with the Kolmogorov-Smirnoff test and Levene’s test for normal distribution.

### Ethical approval:

Local Ethics Committee Approval for this study was obtained from Clinical Research Ethics Committee of Çanakkale Onsekiz Mart University (Decision no: 2023/02-02, decision date: January 18, 2023).

## RESULTS

In our study, 744-HCWs were identified as having been infected with COVID-19 during the 33 months study period. Of the HCWs, 103(14%) were infected in the first 10 months of the pandemic when vaccines were not developed, 641(86%) were infected in the 23 months period after vaccination began. If we exclude the first 10-month period in which there was no vaccine, in the remaining 23 months forty (6.2%) of the 641 patients infected with COVID-19 preferred not to be vaccinated despite the availability of vaccines at our hospital. Table-I

**Table-I:** Characteristics of healthcare workers (HCWs) infected with SARS-CoV-2.

Variable	n(%)
***Age (year)(mean)***	
<26	93(12.5)
26-35	400(53.8)
36-45	190(25.5)
46-55	55(7.4)
>55	6(0.8)
***Gender***	
Male	258(34.7)
Female	486(65.3)
***Occupation***	
Nurse-midwife	215(28.9)
Doctor	192(25.8)
Secretary	70(9.4)
Patient care and cleaning staff	76(10.2)
Others	191(25.9)
***Clinic***	
Asymptomatic	53(7.1)
Symptomatic	691(92.9)
***COVID-19 status***	
diagnosed before vaccination	103(14.0)
diagnosed after vaccination	641(86.0)
***COVID-19 follow-up***	
Outpatient	732(98.4)
Inpatient	10(1.3)
Intensive care unit(ICU)	2(0.3)
***Vaccination type***	143(19.2)
Unvaccinated	15(2.0)
1-shot-vaccinated	292(39.2)
Homologous vaccinated	294(39.5)
Heterologous vaccinated	744(100)
Total	
***Vaccine shots***	143(19.2)
Unvaccinated	15(2.0)
1-shot-vaccinated	206(27.7)
2-shot-vaccinated	165(22.2)
3-shot-vaccinated	215(28.9)
≥3-shot-vaccinated	744(100)
Total	

Homologous: two or more shots of the same vaccine. Heterologous: at least 2 shots of the same vaccine plus 1 or more shots of another vaccine.

Seven percent of patients were asymptomatic. The majority of infected HCWs were women (65.3%). The mean age of the HCWs was 33 years (Table-I) although the rate of those infected in the 26-35 age range was statistically significantly higher (p: 0.0001). Once infected, 1.3% (n=10) and 0.3% (n=2) of the patients required hospitalization and ICU support, respectively; of them, 98.4% (*n=*732) were outpatients. Although infections primarily occurred in women, among the outpatients and the inpatients, the two severe cases admitted to the ICU were men. By occupation, 215 of the HCWs (28.9%) were nurses, and 192 (25.8%) were doctors; of them, 93.3% were symptomatic. Thus, though COVID-19 infections occurred mostly among women and nurses, both ICU patients who were followed up were doctors as well as men. By age, rate of patients 26-35 years old who were infected was the highest by a significant percent (Table-I). HCWs were vaccinated with one to five doses of COVID-19 vaccine(s), and 14 different combinations of vaccines, consisting of mRNA and Sinovac/CoronaVac vaccines, were identified (Table-III). Regarding the vaccine brand preferences of the HCWs, of the 601 vaccinated patients, 39.2%(n=292) were homologously vaccinated, 39.5%(n=294) were heterologously vaccinated, 2%(n=15) were one-shot-vaccinated, 27.7%(n=206) were two-shot-vaccinated, 22.2%(n=165) were three-shot-vaccinated, and 28.9%(n=215) were more than three-shot-vaccinated, respectively, compared with 19.2%(n=143) of the unvaccinated (Table-I). The number of people vaccinated with more than three doses was higher than other groups. By gender, no significant difference in vaccination rates emerged among the infected HCWs (Table-II).

**Table-II:** Comparison of HCWs infected with SARS-CoV-2 by COVID-19 vaccination status.

	Unvaccinated	Vaccinated	P
**Age, *mean***	32.9±8.5	33.0±7.9	0.845
Gender	n(%)	n(%)	
Female	90(18.5)	396(81.5)	0.505
Male	53(20.5)	205(79.5)	
Total	143(19.2)	601(80.2)	

**Table-III:** Distribution of vaccine brands and mean time after vaccination until the onset of infection.

Homologue Vaccinated				Heterologous Vaccinated				Partially vaccinated				

Vaccine Brand	Mean	Std. Deviation	Min-max	Vaccine Brand	Mean	Std. Deviation	Min-max	Vaccine Brand	Mean	Std. Deviation	Min-max	95% Confidence Interval of the Difference**
2-B (n=46)	145.1	66.6	6-365	1-S + 1-B (n=1)	19	19	19	1-B (n=5)	63.4	91.3	2-214	
2-S (n=159)	157.5	123.5	2-539	1-S + 2-B (n=1)	60	60	60	1-S (n=10)	141.3	131.2	20-369	
3-B (n=19)	74.2	76.8	1-257	2-S + 1-B (n=93)	178.1	74.8	2-420					
3-S (n=52)	145.9	83.7	3-381	2-S + 2-B (n=156)	115.8	75.9	1-373					
4-S (n=14)	63.2	40.1	6-160	2-S + 3-B (n=34)	54.9	42.6	4-219					
5-S (n=2)	129.5	74.2	77-182	3-S + 1-B (n=9)	71	69.01	2-239					
Total (n=292)	143.4	106.7	1-539	Total (n=294)	126.4	82.4	1-420	Total (n=15)	115.3	122.1	2-369	1.52 – 32.48

S: Sinovac/CoronaVac. B: BioNTech, *p:0.031 [Comparison of homologous and heterologous groups],

** p:0.250 [Comparison of homologous, heterologous, and partially vaccinated groups].

Our study also showed that the intensity of homologous and heterologous vaccination selection rates was similar among HCWs, and most HCWs (28.9%) had received more than three doses of vaccine(s). The majority of infected HCWs had received the Sinovac/CoronaVac vaccination, as shown in Table-III.

There was a significant difference in the time from vaccination until onset of COVID-19 between HCWs who received homologous versus heterologous vaccines. Heterologously vaccinated HCWs (126.4±82.43) had COVID-19 significantly earlier (*p=0*.031) than homologously vaccinated ones (143.4±106.7), as shown in Table-III. However, no significant difference emerged between the homologous, heterologous, and partly vaccinated groups in terms of the time elapsed from vaccination until the onset of infection (Table-III, *p=0*.250).

Of the 143 unvaccinated infected HCWs, 40(28.0%) were not vaccinated due to vaccine hesitation and 103(72%) due to vaccine shortages. We determined that the remaining 601 (80.8%) infected HCWs had received at least one dose of the vaccine. As a result, of the 641 patients infected during the 23-month period of vaccination, 601(%94) were vaccinated and 40 (%6) were unvaccinated. The large number of people infected despite being vaccinated led us to conclude that the vaccine did not prevent infection but reduces hospitalization. On average, outpatients became infected mean 134(range:1-539), inpatient 79 and ICU patients 229-days after vaccination (Table-IV). Our analysis also revealed that most of the infected HCWs had been vaccinated at least once, whereas only 19% of them were unvaccinated.

**Table-IV:** Mean number of days between the last dose of vaccination and the onset of COVID-19 infection.

	n	Mean	Median	S.D.	Minimum-Maximum	P*
Outpatient	597	134.3	116	95.9	1-539	0.273
Inpatient	2	79.5	79.5	109.6	2-157	
ICU	2	229.5	229.5	202.9	86-373	
Total	601	134.4	116	96.3	1-539	

S.D.: Standard deviation, ICU: Intensive Care Unit,

* Kruskal Wallis Test.

The fact that the number of infected patients despite being vaccinated was higher than that of unvaccinated infected patients led us to conclude that the vaccine does not prevent infection. As shown in Table-II, when infected vaccinated and unvaccinated patients were compared, there was no statistically significant difference in the mean age and gender distribution of both groups.

Although we showed that the vaccine did not reduce the risk of contracting the disease, we concluded that it reduced hospitalization, considering that 80 percent of inpatients were unvaccinated, as seen in Table-V. Of the two male patients in intensive care, one received a single dose of vaccine while the other received two doses. As shown in Table-V, there was a significant difference in the mean age(p=0.009), gender (p=0.039) and vaccination status(<0.001) of inpatients, intensive care and outpatients, respectively.

**Table-V:** Disease course of COVID-19 (*n=*744).

	Outpatient(n=732)	Inpatient(n=10)	ICU patient(n=2)	TOTAL(n=744)	p
Age, mean	32.8±8.0	40.6±8.9	36.0±2.8	33.0±8.0	0.009
	n(%)	n(%)	n(%)	n(%)	
Gender					
Female	477(65.2)	9(90)	0	486(65.3)	0.039
Male	255(34.8)	1(10)	2(100)	258(34.7)	
Vaccination status					
Unvaccinated	135(18.4)	8(80)	0	143(19.2)	<0.001
2-shots	205(28)	0	1(50)	206(27.7)	
3-shots	165(22.5)	0	0	165(22.2)	
+3-shot	213(29.1)	2(20)	0	215(28.9)	
1-shot	14(1.9	0	1(50)	15(2.0)	
TOTAL (n=744)	732(98.4)	10(1.3)	2(0.03)	744(100.0)	

* ICU: Intensive care unit.

When the patients were classified by clinical follow-up, the mean age was 32.8 ± 8.0 years for outpatients, 40.6 ± 8.9 years for patients hospitalized in the ward, and 36.0 ± 2.8 years for patients hospitalized in the ICU. The mean age of two patients admitted to the ICU was significantly less than that of ward-admitted patients (*p=0*.009). By gender, higher infection rates were found among women than among men in the groups except among ICU patients (p =0.039, Table-V). Both young male patients hospitalized in intensive care were physicians working in the Covid-19 service. While one of them had no comorbid diseases, the other had accompanying obesity and hypertension. All patients survived.

## DISCUSSION

This retrospective study at a University Hospital in Turkey analyzed COVID-19 infections among 744 HCWs from April 6, 2020, to November 1, 2022. This study, which includes faculty members, assistant doctors, nurses, and support staff, aims to fill a gap in existing literature by examining vaccination and infection rates among HCWs in Türkiye. The analysis focuses on demographic, clinical, and vaccination characteristics, as well as attitudes towards COVID-19.

A study by Yılmaz et al.^6^ examined the characteristics of HCWs who contracted COVID-19 after receiving the first dose of the CoronaVac vaccine. The study included 4,195 workers, with 3,259 receiving the first dose. Of these, 77.68% were vaccinated. The study found that undetected cases of COVID-19, particularly among healthcare workers, could be dangerous for patients and other healthcare workers. Yılmaz et al. reported that the primary goal should be vaccination of all HCWs, but personal protective measures should also be maintained in the fight against the disease^6^. In our study, it was found that 81% of infected healthcare personnel were vaccinated.

A meta-analysis conducted on COVID-19 infections among HCWs revealed an incidence rate of 9.9% for severe or critical disease, with a mortality rate of 0.3%^7^. In a study by Ujyan et al. conducted in Pakistan, the majority of COVID-19 cases were asymptomatic (90%), followed by symptomatic (7%) and critically ill cases (3%), with a mortality rate of 2.8%.^8^ The male to female ratio was 68.6% in the retrospective analysis conducted by Abbas et al., which comprised 51 fatal cases.^9^ In our study, there was no mortality in infected HCWs. The infection rate was higher in women. While the infection rate in women was higher in Yang’s study of healthcare workers in North America (58.2%), in Sharma’s single-center study in Delhi, the infection rate in men was significantly higher (64.9%).^10^,^11^

This study reveals a gender disparity in COVID-19 outcomes among HCWs, with low hospitalization and ICU admission rates. The absence of fatalities and a high rate of outpatients aligns with the literature, suggesting vaccination significantly reduces the risk of severe disease and hospitalization. However, caution is needed due to the dynamic nature of COVID-19 and ongoing vaccination campaigns. This study provides a comprehensive understanding of the impact of COVID-19 on HCWs, with low hospitalization and absence of fatalities in the vaccinated cohort offering optimism about vaccination’s efficacy in mitigating severe outcomes.

In a systematic review concerning the infection and mortality of HCWs with COVID-19, the overall median age was 47.3 years (range: 18-84), and 71.6% of the HCWs were women. Although infections primarily occurred among women, deaths primarily among men.^12^ In the study of Ujjan et al, city Sukkur and Hyderabad was screened for Corona virus infection and the mean age of overall population was 57.83±8.84.^8^ In our study, women outnumbered men, at a rate comparable to that reported in all studies on HCWs, including ones conducted in China (71.8%)^13^, but diametrically opposed to rates found in Egypt (66.2% men), and Italy (75.0% men).^14^,^15^ This study reveals a gender imbalance in COVID-19 infection rates among women, particularly in nursing. This may be due to the close patient interaction and exposure to infectious agents in nursing roles. Societal and occupational factors may also contribute to the gender disproportion. Understanding these gender disparities is crucial for designing preventive strategies and workplace interventions. Future research could explore the dynamics of gender and occupational risk factors to inform evidence-based interventions and policies to mitigate COVID-19 transmission in healthcare settings.

Among fully vaccinated HCWs across 16 studies and unvaccinated HCWs across eight studies, the overall pooled proportions of COVID-19 infections were 1.3% and 10.1%, respectively. Meanwhile, the overall pooled proportions of both fully and partly vaccinated HCWs hospitalized, requiring admission to the ICU, and HCWs dying from COVID-19 infection were 5.7% (95%CI 3.5-9.1; I^2^ 48.4%), 2.6% (95%CI 0.4-15.4; I^2^ 84%), and 1.2% (95% CI 0.3-5.7; I^2^ 72.6%).^16^ In another study, Au et al. assessed the effectiveness of heterologous and homologous COVID-19 vaccine regimens and found that a three-dose mRNA regimen was the most effective against asymptomatic and symptomatic COVID-19 infections.^17^ Concerning time from vaccination to onset, Amit et al. found that 22 of 4,081 vaccinated HCWs (0.54%) developed COVID-19 in 1-10-days (median:3.5-days) after immunization in Israel.^18^ In our study, 24 of 744 HCWs developed COVID-19 in 1-10-days (median:4.5-days) after the first dose of vaccination. The incidence of COVID-19 after vaccination is similar between the two studies, despite differences in the number of vaccinated healthcare workers and the type of vaccine used. Various researchers around the world have investigated the efficacy of COVID-19 vaccines and the length of their protection against SARS-CoV-2 infection. After 196-days of homologous primary immunization, the BNT162b2 and Moderna mRNA vaccines prevented COVID-19 by approximately 67% and 80%, respectively.^19^ The Ad26.COV2.S vaccine was 59% effective after a single dose was administered after 140-days, whereas the ChAdOx1 vaccine was approximately 60% effective when a second dose was administered after 90-days.^20^

In a study conducted between January and December 2021, during the global outbreak of the Omicron variant, the cumulative median protection period following the final dosage of various homologous and heterologous vaccine batches for all primary COVID-19 immunizations was 134-days. Compared with a homologous primary vaccine that included both BNT162b2 and ChAdOx1 and heterologous primary immunization, the heterologous vaccination (i.e., ChAdOx1 followed by BNT162b2) demonstrated a significantly longer median protective duration than BNT162b2 followed by ChAdOx1 (p<0.001). That outcome was consistent with the findings of Mayr et al., who discovered that a heterologous regimen of Ad26.COV2.S followed by Moderna’s COVID-19 vaccine was 56.7% more successful than a homologous vaccination for Ad26.COV2.S and even lasted up to 120-days.^21^ In a recent study, the ChAdOx1 and BNT162b2 COVID-19 primary vaccination regimens given in Saudi Arabia in 2021 had a median protective duration of 134-days. The study also showed that heterologous primary vaccinations (i.e., ChAdOx1 and BNT162b2) had a significantly longer protective duration than other vaccination regimens.^22^

In other research, a robust humoral response was elicited by both the third dose of the vaccine and the heterologous prime-boost immunization. People who had previously contracted the disease and received a single dose of the vaccine had a neutralizing response equal to that of people who received two doses of the vaccine against all SARS-CoV-2 variants.^23^ A study conducted on 103 HCWs in Germany found that a heterologous vector-mRNA-based SARS-CoV-2 vaccination approach seems to be more effective than a homologous vector-based plan. In this study, long-term humoral immunity was found to be sustained, and anti-SARS-CoV-2 anti-N- and anti-RBD/S1-Ig levels were evaluated. After the second vaccination, this study also discovered higher levels for heterologous mRNA/vector combinations than for pure vector-based vaccination. In a cohort with high exposure, the incidence of vaccine breakthrough was 60.3%. The humoral vaccination response and adverse effects were not found to be correlated in the study, and vaccine breakthroughs only happened later in the investigation when there were more infectious variants. The findings shed light on serologic reactions linked to vaccinations and point to potential future expansion using higher vaccine doses and innovative variants.^24^ In our study, all patients became infected at an average of 134-days (range: 1-539) after vaccination and we detected that most infected HCWs were vaccinated at least once, whereas only 19% were unvaccinated. The interval between immunization and the beginning of infection varied significantly between homologous and heterologous vaccinees; homologous vaccinees were infected 143.4±106.7-days later than heterologous ones (126.4±82.4-days). However, the interval from immunization to disease onset did not significantly differ between the homologously, heterologously, and partially vaccinated groups.

### Limitations:

Anti-SARS-COV-2 antibody titers could not be measured, which limited us from investigating their relationship to vaccine efficacy. Another one is that, considering the examined period, various variants of Sars-Cov2 followed each other over time and because they had different pathogenicity within themselves, the vaccination strategy changed dynamically, and we could not access data on this change during the study.

## CONCLUSION

In this study, the fact that the number of infected patients despite being vaccinated was higher than the unvaccinated infected patients led us to the conclusion that the vaccine did not prevent infection but reduced hospitalizations. In the first 10 months of the epidemic, when there was no vaccine but quarantine measures were strictly implemented, the number of HCWs infected was 103, while 641 people were infected during the period when ongoing vaccination was carried out but quarantine measures were relaxed. This result made us think that quarantine measures made a more significant difference than vaccination in preventing the spread of the epidemic.

By vaccination status, 39.2% of the HCWs were homologously vaccinated, 39.5% heterologously vaccinated, and 19.2% unvaccinated. Homologously vaccinated HCWs contracted COVID-19 later than heterologously vaccinated ones, and most unvaccinated HCWs did not receive the vaccine due to vaccine hesitation or vaccine shortages.

### Authors’ Contribution:

**IDA:** Conceptualization, data curation, formal analysis, methodology, writing - original draft, writing - review & editing.

**YHE:** Data curation, software, writing - original draft, writing - review & editing and also responsible for the accuracy of the work.

**GC:** Data curation, formal analysis, writing - review & editing.

**SA:** Conceptualization, data curation, writing - original draft, writing - review & editing.
